# Recurrent gastric antral vascular ectasia: a single center experience

**DOI:** 10.3389/fsurg.2024.1356409

**Published:** 2024-04-03

**Authors:** Andrea Cavallaro, Antonio Zanghì, Maria Di Vita, Vito Emanuele Catania, Giovanni Longo, Emanuele Lo Menzo, Roberta Granata, Maria Rosaria Valenti, Alessandro Cappellani, Simone Di Majo

**Affiliations:** ^1^ General Surgery III, Department of Surgery, University of Catania, AOU Policlinico “G. Rodolico - San Marco,” Catania, Italy; ^2^ Department of Medical, Surgical Sciences and Advanced Technologies G.F. Igrassia, Department of Catania, Chief ChiSMaCoTA Research Center, AOU Policlinico “G. Rodolico - San Marco,” Catania, Italy; ^3^ Department of General Surgery, Division of Minimally Invasive and Metabolic Surgery, Cleveland Clinic Florida, Weston, FL, United States

**Keywords:** antral vascular ectasia, GAVE, treatment, APC, RFA, surgery

## Abstract

**Introduction:**

Gastric antral vascular ectasia (GAVE) is a rare cause of chronic or acute gastrointestinal bleeding. This condition accounts for ∼4% of upper gastrointestinal bleeding cases. This disease is often associated with systemic diseases, such as liver cirrhosis, chronic kidney failure, autoimmune conditions, diabetes mellitus, hypothyroidism, and cardiovascular diseases. However, its etiopathogenesis remains controversial.

**Materials and method:**

We retrospectively reviewed the cases of GAVE treated at our digestive surgery unit. A total of nine patients were identified with a male/female ratio of 1.25:1 and an average age of 75.51 years (*SD *± 9.85). All patients underwent endoscopic argon plasma coagulation (APC) treatment. At the time of the review, data on eight patients were available after 36 months of follow-up.

**Results:**

APC appears to be safe and effective for hemostasis of bleeding vascular ectasia. Only one (11.1%) patient required surgical intervention due to hemodynamic instability after multiple unsuccessful endoscopic treatments. No intraoperative and postoperative complication or bleeding relapse was experienced.

**Discussion:**

Based on our findings, we concluded that endoscopic APC is technically simple, but requires multiple re-interventions due to the incidence of relapses. Furthermore, larger randomized studies should be conducted to assess the role of elective surgery as the first intervention in stable patients with severe pathology and the timing of surgery after failed endoscopic treatment.

## Introduction

Acute upper gastrointestinal hemorrhage (UGIH) is a widespread condition with an estimated global annual incidence of 40–150 cases per 100,000 individuals, which varies slightly between countries. Acute UGIH frequently leads to hospitalization and is associated with high morbidity and mortality, especially in the elderly (1).

The most common causes of UGIH are not associated with esophageal varices and include:

- Peptic ulcers, 28%–59% (duodenal, 17%–37%; gastric, 11%–24%)
- Erosive disease of the esophagus/stomach/duodenum, 1%–47%
- Mallory–Weiss syndrome, 4%–7%
- Malignant tumors of the upper GI tract, 2%–4%
- Other diagnoses, 2%–7%
- Unknown etiology, 7%–25%

Gastric antral vascular ectasia (GAVE), also known as “watermelon stomach,” is a rarer cause of acute UGIH. This condition accounts for ∼4% of upper gastrointestinal bleeding cases and is characterized by the presence of columns of vessels in the antral longitudinal folds converging into the pylorus (2–4).

The hyperplasia of the mucosa, with abnormal vessels in the submucosa and the fibromuscular hyperplasia of the lamina propria, leads to capillary dilation and intravasal thrombosis.

The proposed pathophysiology has been mechanical stress, hypergastrinemia, associated hormonal imbalance or vasoactive mediators, abnormal antral motility, or autoimmune connective tissue disease associated with vasculopathy and gastrointestinal tract manifestation (5).

Medical therapies have been ineffective and are mainly limited to experimental studies. Currently, the endoscopic argon plasma coagulation (APC) approach is considered the first line of treatment. Although generally effective, patients need repeated sessions.

Surgery is considered the last therapeutic option for hemodynamically unstable patients and refractory cases, as GAVE patients are usually poor surgical candidates due to their comorbidities. We present our experience in the treatment of this condition.

## Materials and methods

After receiving hospital ethical board approval, we retrospectively reviewed all cases of GAVE treated between 2009 and 2019 at the Gastroenterology and Digestive Endoscopy Unit and the General Surgery and Senology Unit of the Department of Surgery, Policlinico “G. Rodolico–San Marco” Hospital.

The study included all patients with endoscopic diagnosis of GAVE.

Biopsy data were used alongside visual findings to confirm the diagnosis in applicable clinical settings.

Patients without GAVE and those with other types of upper gastrointestinal bleeding were excluded.

All data were collected from medical records and stored in a secure de-identified spreadsheet.

Baseline demographic data, such as age, sex, BMI comorbidities, and clinical and endoscopic characteristics, were collected and analyzed from all patients.

The number of endoscopic interventions, inpatient vs. outpatient procedures, and hemoglobin levels before EGDS were also recorded.

The overall nutritional and metabolic status of the patient was evaluated based on fasting laboratory values.

We used plasma obtained from the blood samples added with EDTA and centrifuged at 3,000×*g* for 15 min at 4°C. Immediately after centrifugation, the plasma samples were frozen and stored at −80°C. The total cholesterol and triglycerides were determined using fully enzymatic techniques on a clinical chemistry analyzer; the intra- and interassay CVs were 1.1% and 2.2% for the total cholesterol measurement and 1.0% and 2.3% for the triglyceride measurement, respectively.

The HDL cholesterol level was measured after the precipitation of plasma apo-B-containing lipoproteins with phosphotungstic acid. The intra- and interassay CVs were 1.0% and 2.0%, respectively. The LDL-C level was calculated using the Friedewald formula.

Serum Hcy and vitamin B6 were quantified after protein precipitation and derivation using isocratic high-performance liquid chromatography with fluorescence detection, on an Agilent chromatography system (Chromsystem Instruments & Chemicals Gmbh, Germany).

The serum level of vitamin B12 was measured on an Immunolite 1000 analyzer with a specific ELISA kit (PDC, Siemens, Los Angeles, CA, USA), according to the manufacturer's instructions. The serum level of folate was measured using Quantaphaseradioassay (Bio-Rad Laboratories) (6).

## Results

A total of nine patients were identified during the study period, four females and five males (male:/female ratio, 1.25:1). The *mean age* at the time of the diagnosis was 75.51 years (*SD *± 9.85).

Of the nine patients, five had anemia and melena (55.5%), three had indeterminate dyspepsia (33.3%), and one was asymptomatic (incidental diagnosis).

The following comorbidities were present at the time of the diagnosis of GAVE: four patients had chronic hepatitis, two of whom had liver cirrhosis and esophageal varices, one patient had chronic hepatitis and kidney failure, two had isolated chronic kidney failure at an advanced stage, and two had no comorbidities.

All patients at the time of diagnosis demonstrated platelet count above 100,000 (platelets per microliter), an INR value within the normal range. Seven patients demonstrated low hemoglobin and hematocrit, and all nine patients had low iron and transferrin levels.

Aspecific deficiencies in vitamin D, folate, carnitine, vitamin B12, or iron were detected. Five patients demonstrated an increase in homocysteine.

**Figure 1:**
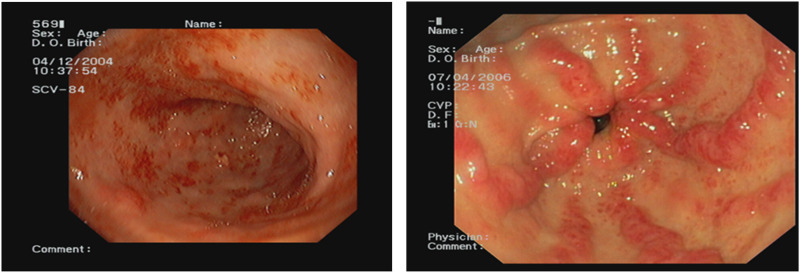
Endoscopic view of the two types of gastric antral vascular ectasia (GAVE) most encountered in our series: petechial appearance and classic striped or “watermelon stomach.”

The endoscopic appearance ranged from petechial (*n* = 5, 55.5%) to classic striped (*n* = 3, 33.3%). One patient demonstrated the uncommon nodular variant (Figure 1).

Multiple biopsies were performed alongside visual inspection to confirm the diagnosis. Only six out of nine patients demonstrated a GAVE score ≥3 (fibrohyalinosis, spindle cell proliferation, or vascular ectasia/thrombi).

In six out of nine patients, computer tomography and ultrasonography of the abdomen revealed hepatomegaly.

Given the high probability of medical therapy failure, the endoscopic approach was the first line of treatment strategy for all patients. Only two patients underwent initial medical treatment with oral octreotide 20 mg, without clear benefits. Eight patients were treated with proton pump inhibitors in between sessions of APC.

One patient required surgical intervention after several sessions of APC and multiple blood transfusions.

**Figure 2:**
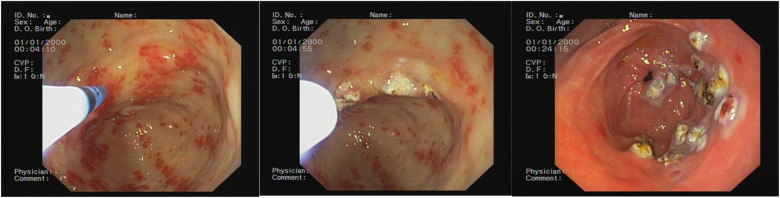
Endoscopic appearance before the argon plasma coagulation (APC) treatment, during the APC treatment, and after the APC treatment.

**Figure 3:**
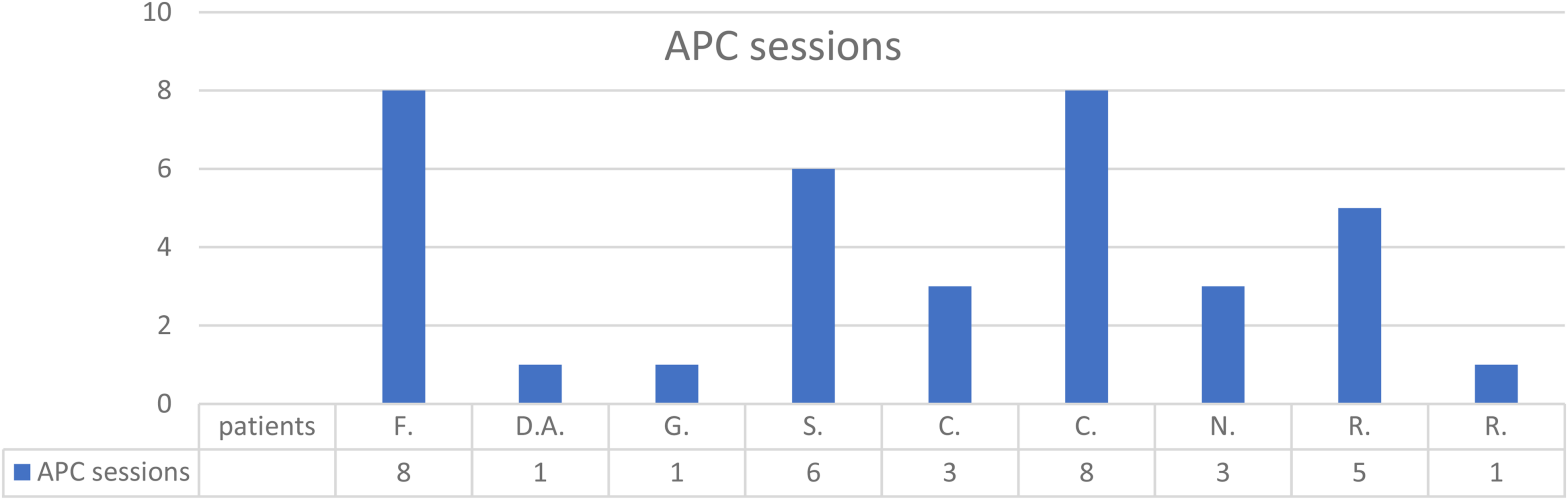
Number of endoscopic sessions per patient.

The average number of endoscopic APC sessions (Figures 2, 3) was 4 (1–8 sessions, SD: 2.7), with a mean timeframe between consecutive sessions of 3 months (19 days–21 months). Over time, there was a progressive reduction of the time between each procedure.

**Figure 4:**
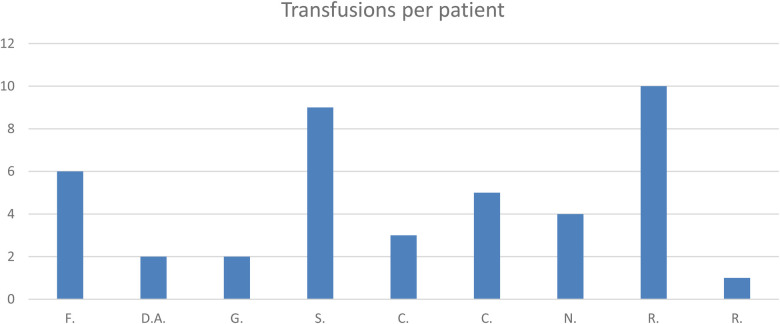
Number of transfusions per patient.

Only eight patients were available for follow-up (36 months). Seven patients required endoscopic treatment combined with medical therapy. One patient failed to respond to endoscopic treatment and was referred for surgery. The patients treated endoscopically suffered from chronic/recurrent sideropenic anemia. Six of them required further endoscopic inspections, and all of them underwent blood transfusions (average, 4.66 units; SD, 2.98) (Figure 4).

Because of hemodynamic instability (after the failure of five sessions of APC), after 10 units of blood transfusions, and the failure of interventional radiology embolization, one patient underwent open distal gastrectomy with Roux-en-Y reconstruction. There were no postoperative complications, and the patient experienced complete resolution of the gastrointestinal bleeding.

## Discussion

GAVE is a rare acquired vascular disease accounting for ∼4% of upper gastrointestinal bleeding cases.

The disease was first described by Rider et al. (3) in 1953 in patients with chronic anemia.

Later, in 1984, Jabbari et al. (4) characterized the disease more accurately with the description of longitudinal antral plicae that converge toward the pylorus; hence, they named the condition “watermelon stomach.”

The etiopathogenesis has not been fully identified, so it remains controversial.

The disease is often associated with systemic diseases, such as liver cirrhosis, chronic kidney failure, autoimmune conditions, diabetes mellitus, hypothyroidism, and cardiovascular diseases. The disease is more prevalent in women with an average age of 80 years (7–10).

Up to 30% of GAVE patients present liver cirrhosis, and 1 in 40 patients with a history of liver transplant present the disease. In the case of liver disease-associated GAVE, the patients are more often males with a lower average age of onset (65 years) (7).

However, GAVE remains a rarer cause of bleeding in cirrhotic patients, as hypertensive gastropathy (PHG) is more frequently reported.

In the subpopulation of non-cirrhotic patients, GAVE is associated with autoimmune pathologies, especially connective disorders in 62% of cases, Raynaud's disease in 31%, sclerodactyly in 20%, and, more rarely, systemic sclerosis in 5.7%–22.3% (11, 12).

The clinical presentation widely ranges from occult and asymptomatic blood loss associated with chronic iron deficiency anemia up to hematemesis, melena, or, even, rectorrhagia and abdominal pain.

In its acute presentation, GAVE could be associated with syncope, tachycardia, orthostatic hypotension, hemodynamic instability, and hypovolemic shock (13).

In the literature, the need for transfusions has been reported in more than 62% of the patients (8, 9).

In rare cases, GAVE may present with occasional abdominal pain or even gastric outlet obstruction (14).

The diagnosis is often made endoscopically, with the presence of strips of red spots in the antrum or a diffuse honeycomb appearance. Less common patterns are petechial appearance, the nodular ones, and the slightly elevated shape “mushroom” like pattern (15).

The biopsy is often necessary to differentiate GAVE from pathologies such as hypertensive gastropathy (15–19).

Histologically the tortuous ectasic vessels of vascular ectasia extend over the submucosal layer (15).

In 1989, Gilliam et al. proposed a scoring system for the diagnosis of GAVE based on two histological criteria: the coexistence of ectasia, the presence of fibrin thrombi, and/or fusiform cell proliferation (Gilliam score) (12, 13).

Subsequently, fibroialinosis was added as a third criterion to improve the sensitivity and specificity of the diagnosis. The so-called GAVE score has high diagnostic accuracy, especially in differentiating GAVE from hypertensive gastropathy (20).

The main goal of the treatment of GAVE remains the control of gastrointestinal hemorrhage.

The first line of treatment in any severe gastrointestinal bleed remains supportive with appropriate fluid and blood resuscitation.

Several pharmacological strategies have been studied, but none have presented satisfactory results. Among the proposed drugs utilized in the medical treatment of GAVE are proton pump inhibitors, tranexamic acid, octreotide (0.1 mg 2–3 times daily or 20 mg monthly), cyproheptadine (for patients with elevated serotonin concentration), cyclosporine, methyl prednisone (in cases related to autoimmune diseases), cyclophosphamide (suitable for patients with systemic sclerosis only), calcitonin (10 units intramuscularly twice a week in osteoporotic patients), thalidomide (for patients on anticoagulant or antiplatelet drugs), and bevacizumab (21, 22).

The most common therapeutic approach remains the upper endoscopy with several types of interventions, such as heater probes, Nd:YAG laser, ligature, cryotherapy, radiofrequency ablation (RFA), and APC (23–25).

APC is characterized by non-contact coagulation, determined by high-frequency monopolar current through ionized and electrically conductive argon gas. This technique allows tangential application and thus treatment of the target site in a uniform manner to a depth of approximately 1–3 mm, which is useful to coagulate the superficial blood vessel. The coagulation depth of APC depends on the power generator settings, distance of the target tissue, and duration of the application (26).

Because of the ability to safely cauterize large surfaces with minimal depth penetration, APC is considered to be the first choice. The most common setting of APC uses 20–80 W of electricity with 0.5–2 L/min of argon gas flow.

Large case series using APC have reported an efficacy ranging from 90% to 100%, with no further need for blood transfusions and a mean increase in hemoglobin levels by 3 g/dl (27–29).

However, because the APC is used to treat only the gastric surface, this modality does not present satisfactory long-term results. Consequently, this approach almost always requires multiple sessions every 2–6 weeks. Patients treated with this technique present a recurrence of the bleeding in up to 78.9% of the cases (27–29).

A second endoscopic treatment modality is RFA.

Although the available evidence suggests that RFA has a comparable efficacy and tolerability compared with APC, long-term data is limited.

In addition, RFA has been reported to be effective for APC refractory GAVE patients (30, 31).

Recently, some studies compared the efficacy of endoscopic band ligation and endoscopic thermal therapy by APC. A meta-analysis of over 507 patients in five studies concluded that endoscopic band ligation led to significantly lower transfusion requirements and showed a trend toward more remarkable postprocedural hemoglobin elevation and a smaller number of procedures. Endoscopic band ligation may improve outcomes and lead to decreased healthcare burden and costs (32, 33).

Surgery for GAVE treatment, although not often reported in the literature, is the definitive treatment in cases of endoscopic failures, refractory patients, and severe hemodynamic instability.

The extent of the surgical resection depends on the extension of the disease, but antrectomy is sufficient in controlling the disease. Occasionally more extensive resections, such as total/subtotal gastrectomy, are necessary. The surgical approach can be either open or laparoscopic depending on the clinical condition of the patient and the surgeon’s experience (34, 35).

The reported mortality after surgical intervention ranges between 6.6% and 7.4% (34, 35).

The higher morbidity and mortality of the surgical approach compared to the endoscopy treatment is likely due to the coexisting comorbidities of patients, such as liver failure, cirrhosis, and portal hypertension (36–38).

Common complications described after gastrectomy for GAVE are dumping syndrome, weight loss, and vitamin and mineral deficiencies (iron, calcium, and vitamins B12 and D in particular) (39–43).

Cases of heterotopic mesenteric ossification after abdominal operation for recurrent gastric bleeding due to GAVE have been also reported (44).

While a significant amount of research is needed to fully understand GAVE syndrome and its optimal management, surgical resection may prove to be a viable option in refractory patient populations with severe recurrent bleeding and failed medical management or inpatients requiring anticoagulation (45–48).

An earlier surgical approach could be utilized, in some selected cases requiring long-term anticoagulation and dual antiplatelet therapy or in patients with low compliance to transfusion dependency and elevated frequency of readmissions, as a definitive intervention. This approach could reduce the number of admissions of patients in hospitals and at the same time hospitalization costs (45–48).

Several flowchart diagrams for GAVE treatment have been proposed over time in the literature to select the best endoscopic treatment based on the macroscopic pattern, size, and spreading of the lesions. Some authors proposed a cutoff value of 3–4 unsuccessful endoscopic attempts to select other therapeutic strategies in a step-up approach: surgery is generally indicated as the last viable option in cases of refractory patients (42, 43).

However, none of these flowcharts has gained wide consensus. Moreover, further randomized controlled studies are awaited, particularly regarding interventional comparisons of APC, RFA, endoscopic banding ligation, and other evolving modalities (49).

In our limited case series, the endoscopic strategies, whose biggest limitation remains the incidence of relapses, showed efficacy in all cases except one who required urgent surgery.

Given the lacking of clear indications in the literature, we prefer a “tailored” approach that takes into account the clinical conditions, the evolution of the pathological process over time, the compliance of the patient with hospital readmissions and/or transfusion dependency, the previous occurrence of life-threatening complications, and the patient's disposition toward surgical treatment.

## Conclusion

GAVE is a rare cause of bleeding in elderly patients with liver disease or other comorbid conditions. These patients are usually not eligible for surgical intervention as the first approach.

In cases of bleeding due to GAVE, the preferred therapeutic approach is endoscopic ablation. This represents a less invasive, but less frequently definitive, approach.

The advantages of endoscopic APC are the technical simplicity, the higher effectiveness in a short time, and the reduced postprocedural complications. Adjustment of the distance between APC probe and the target lesion and the duration of application can achieve a satisfactory effect in a variety of settings. However, most patients treated with such a modality require multiple sessions due to bleeding relapse and ongoing transfusion requirements.

Patients who undergo surgery are more likely to experience a definitive resolution of the pathology.

However, the clinical question related to the surgical approach as the first line of treatment, or treatment of recurrent/refractory cases after a certain number of endoscopic sessions, remains to be determined in larger randomized trials.

## Data Availability

The raw data supporting the conclusions of this article will be made available by the authors, without undue reservation.
